# Cytotoxicity of Environmentally Relevant Concentrations of Aluminum in Murine Thymocytes and Lymphocytes

**DOI:** 10.1155/2011/796719

**Published:** 2011-06-27

**Authors:** Jamal Kamalov, David O. Carpenter, Irina Birman

**Affiliations:** ^1^School of Public Health, University at Albany, Rensselaer, NY 12144, USA; ^2^Institute for Health and the Environment, University at Albany, Rensselaer, NY 12144, USA

## Abstract

The effects of low concentrations of aluminum chloride on thymocytes and lymphocytes acutely dissociated from young mice were studied using flow cytometry with a DNA-binding dye. We demonstrate a rapid and dose-dependent injury in murine thymocytes and lymphocytes resulting from exposure to aluminum, as indicated by an increase in the entry into the cell of the DNA-binding dye, propidium iodine. A 60-minute exposure to 10 *μ*M AlCl_3_ caused damage of about 5% of thymocytes, while 50% were injured after 10 minutes at 20 *μ*M. Nearly all thymocytes showed evidence of damage at 30 *μ*M AlCl_3_ after only 5 minutes of incubation. In lymphocytes, injury was observed at 15 *μ*M AlCl_3_ and less than 50% of cells were injured after a 60-minute exposure to 20 *μ*M. Injury only rarely proceeded to rapid cell death and was associated with cell swelling. These results suggest that aluminum has cytotoxic effects on cells of the immune system.

## 1. Introduction

Aluminum is one of the most abundant elements on earth but has no known biological function in living organisms [1]. Exposure to aluminum and its associated toxicity are well documented in plants and animals [2, 3]. In humans, aluminum toxicity was first described as osteomalacic dialysis osteodystrophy [4]. Although aluminum has been primarily recognized as a neurotoxin and etiologic agent of dialysis dementia [5, 6], other detrimental health effects have been documented [7]. Some authors report aluminum-induced genotoxicity [8]. Others associate exposure to aluminum with osteodystrophy [9], anemia [10], and altered calcium homeostasis [11]. In addition, underlying conditions such as renal failure, leukemia, and diabetes increase aluminum retention in human and animal subjects due to impaired absorption and excretion, which in turn exacerbates its toxic effect [12, 13]. 

Evidence regarding the effect of aluminum on the immune system is limited and conflicting. Some researchers report immunosuppression, while others portray aluminum as an efficient adjuvant in vaccines [14, 15]. Exposure to low concentrations of aluminum was reported to cause immunopotentiating effects, whereas exposure to high levels caused immunosuppression [16, 17]. Some authors report that long-term exposure to low concentrations of aluminum resulted in elevated intracellular levels in lymphocytes, which might be a contributing factor in the reported immunosuppression [18]. However, none of this evidence is very convincing in the absence of clear understanding of the mechanism(s) of immunotoxicity [19]. 

Human exposure to aluminum other than during dialysis occurs primarily through ingestion of food and water, utilization of personal care products and cookware, and consumption of medications and administered vaccines [20–22]. Elevated levels of aluminum in soils have been implicated in the higher frequency of neurodegenerative disorders in the Kii Peninsula and natives in Guam [23].

 Due to rapid urbanization, anthropogenic contaminants accumulate in various environmental media, including source water and sediment, food and pharmaceuticals, air, and dust [24, 25]. While numerous studies have been conducted on aluminum toxicity, nearly all of them have investigated effects of exposure to high concentrations. These concentrations are not representative of typical environmental exposure levels and cannot be associated with ordinary circumstances for people with normal renal function.

Due to its abundance in nature and in man-made products, cumulative daily uptake of aluminum by humans is difficult to estimate. Study of aluminum is further complicated by the fact that a variety of complexes are formed in solution [26], and these various forms may have different toxicities and biological effects. Based on available information, we have attempted to estimate the range of daily exposure in humans and study the effects of AlCl_3_ solutions on isolated immune system cells at concentrations that are environmentally relevant. In the present study, we have investigated the effects of low concentrations of aluminum on thymocytes and lymphocytes that were acutely dissociated from young mice. Our data indicate that exposure to aluminum results in a dose- and time-dependent damage of the plasma membrane of thymocytes and lymphocytes but does not cause acute cell death to any significant degree.

## 2. Materials and Methods

### 2.1. Reagents

Aluminum chloride (III) of 99.95% purity grade was purchased from Sigma-Aldrich (St. Louis, Mo, USA) and was dissolved in distilled water. Propidium iodide (PI) and Annexin V-FITS apoptosis detection kit were purchased from Sigma-Aldrich (St. Louis, Mo, USA).

### 2.2. Preparation of Thymocytes and Lymphocytes

These investigations were reviewed and approved by the University at Albany Animal Care and Use Committee. Thymocytes were acutely dissociated from the thymus gland of four-week-old ICR male mice (Taconic Biotechnology, Inc., Germantown, NY) as previously described [27], while lymphocytes were separated from the spleen. Mice were rapidly decapitated with a guillotine, and the thymus and spleen were removed. To obtain cell suspensions, the organs were gently ground between frosted glass microscope slides. Red blood cells in spleen tissue were lysed with RBC-lysing buffer (0.15 M NH_4_Cl, 10 mM KHCO_3_, and 0.1 mM Na_2_EDTA, pH 7.2–7.4). The suspensions of remaining white cells were then filtered by gravity through a cell strainer (70 *μ*m) to obtain a more uniform single-cell suspension. Unless otherwise specified, all experiments were conducted at 37°C with freshly prepared Tyrode's solution (148 mM NaCl, 5 mM KCl, 2 mM CaCl_2_, 1 mM MgCl_2_, 10 mM glucose, 10 mM HEPES, pH 7.4). The strained suspensions were washed three times with Tyrode's solution. Before being loaded with fluorescent dyes and exposed to AlCl_3_, murine thymocytes or lymphocytes were incubated in Tyrode's for 30 minutes to recover from injury of dissociation.

### 2.3. Loading Thymocytes with Dyes

The viability of thymocytes was determined using propidium iodide (PI), a DNA-binding probe that enters the cell only if the plasma membrane is damaged. This dye was added to a sample tube containing approximately 2 × 10^6^ cells 5 minutes prior to a measurement. The effect of immediate exposure of thymocytes and lymphocytes to aluminum was assessed at 0 time, when cells were first preloaded with PI and then analyzed immediately after addition of AlCl_3_. To distinguish necrosis from apoptosis, we used PI and the Annexin V-FITS apoptosis detection kit. Annexin binds to phosphatidyl serine, which in healthy cells is found only on the inner membrane leaflet but moves to the outer leaflet early in the process of apoptosis. Necrosis, unlike apoptosis, is accompanied by cell swelling and is not associated with movement of phosphatidyl serine. Five thousand cells per sample were analyzed using a BD LSRII flow cytometer. The obtained data were processed via utilization of BD FACSDiva software.

### 2.4. Statistical Analysis

Experimental data values were obtained from at least six independent measurements and are presented as mean ± standard deviation of the mean. Statistical analysis was performed using the Student's paired *t*-test and two-way ANOVA, and a *P* value of < 0.05 was considered significant.

## 3. Results

**Figure 1:**
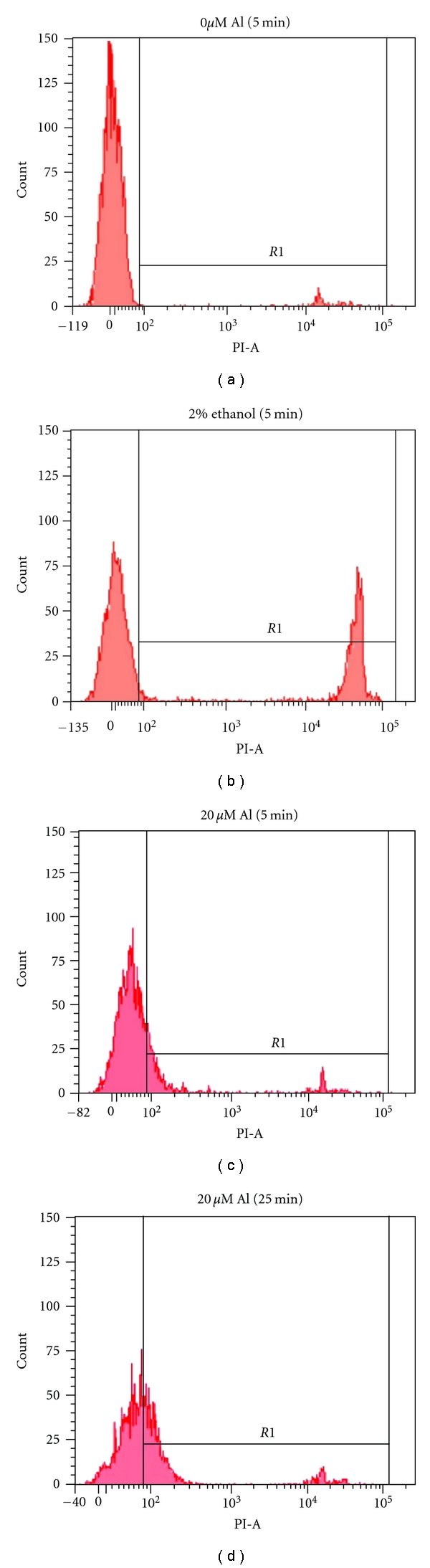
Histograms showing the effects of aluminum exposure to thymocytes. PI fluorescence intensity (*x*-axis) is plotted against cell count (*y*-axis). Histogram (a) shows untreated thymocytes where most of the cells have low PI intensity, which is characteristic of healthy cells whose membranes exclude PI. In (b) thymocytes were exposed to 2% ethanol, and a large number of cells show very high PI intensity. These are dead cells, whose plasma membrane has lost integrity. (c) and (d) show the gradual increase in PI intensity in thymocytes exposed to 20 *μ*M of AlCl_3_ at five- and 25-minute exposure. The number of dead cells did not increase with AlCl_3_ exposure. There is, however, a shift in the distribution of healthy cells to the right, indicating an increased uptake of PI, reflecting cell damage. For purposes of quantitation, all cells falling under the bar labeled R1 are considered injured.

Figure 1 shows how cell injury was detected in our experiments. Histogram A shows untreated cells where a gate (R1) was selected to differentiate between healthy and injured cells. In conventional practice the term “cell death” is used when the PI fluorescence intensity is more than two decades brighter than unstained cells [28]. This is shown in histogram 1(b), where 2% ethanol caused significant thymocyte cell death. Exposure to aluminum resulted in a gradual increase in PI intensity (Figures 1(c) and 1(d)) but did not result in the large increase associated with dead cells. This indicates that aluminum has resulted in some leakage of PI through the plasma membrane, but not to the degree that is seen when membrane integrity is totally lost and cells are dead. Thus we used the term “damage” or “injury” to describe changes associated with aluminum toxicity. All cells in area R1 were considered to be damaged throughout the experiments reported here. 

**Figure 2:**
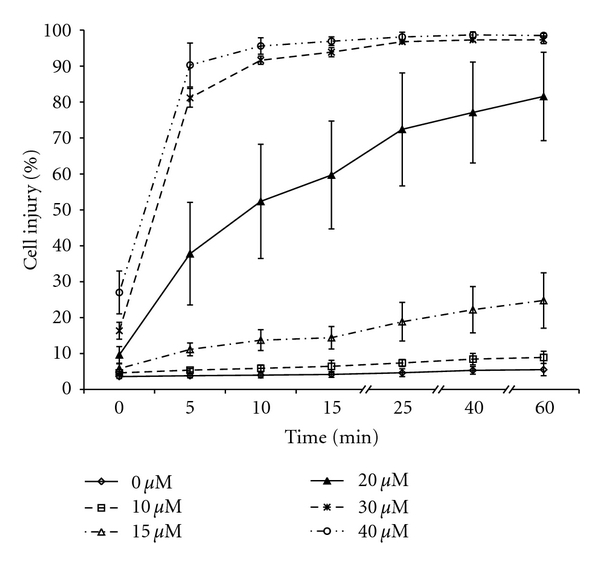
Dose and time dependence of AlCl_3_-induced injury in thymocytes. The cells were treated with a range of concentrations of AlCl_3_ (0–40 *μ*M) at various time points. Thymocytes were considered to be damaged when the level of fluorescence intensity of PI in the cells was higher than the level in untreated cells. Values are mean ± SD obtained from six independent measurements (based on Student's paired *t*-test). The concentration curves are all statistically significantly different at the *P* < .05 level by ANOVA analysis. There are no significant changes with time between 25, 40, and 60 minutes, nor between 10 and 15 minutes, but all other time differences are significant.

Figure 2 shows the dose and time dependence of damage induced by AlCl_3_ in thymocytes. Cell injury was rapid and took place within minutes. We observed significant injury as quickly as the measurements would be taken after exposure to 10 *μ*M AlCl_3_. Cellular damage increased with concentration and exposure time, and after a 10-minute exposure to 20 *μ*M AlCl_3_ close to 50% of the cells showed injury. Nearly all cells were damaged at concentrations of 30 and 40 *μ*M AlCl_3_ after only 5 minutes of incubation (Figure 2). The curve showing cell damage as a function of concentration reached a plateau after 10 minutes. 

**Figure 3:**
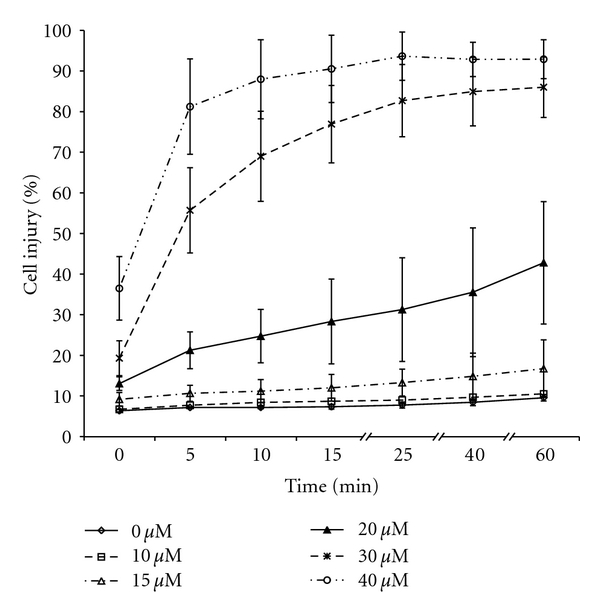
Dose and time dependence of lymphocyte injury with exposure to various concentrations of AlCl_3_ (0–40 *μ*M) at various time points. Other conditions were as described in the legend to Figure 2. All concentration curves are significantly different from each other at the *P* < .05 level by ANOVA with the exception of 0 and 10 minutes. There were no significant changes with time between 25, 40, and 60 minutes, nor between 10 and 15 minutes, but all other time periods were significantly different at the *P* < .05 level by ANOVA.

A similar pattern of injury was observed with lymphocytes (Figure 3), although they were somewhat less sensitive to aluminum toxicity. Significant lymphocyte injury was observed only at a concentration 15 *μ*M, as compared with 10 *μ*M for thymocytes. Less than 50% of lymphocytes incubated with 20 *μ*M AlCl_3_ for 60 minutes were damaged. Moreover, the damaging effect of 30 *μ*M AlCl_3_ on lymphocytes was less pronounced and did not reach the plateau until after 25 minutes of exposure. Our data suggest that lymphocytes exposed to aluminum are less sensitive than thymocytes. 

**Figure 4:**
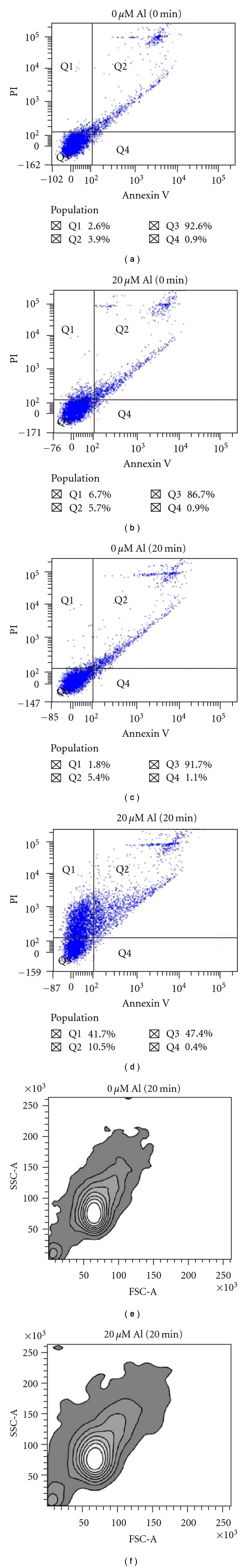
Thymocytes were exposed to 0 *μ*M (a, c) and 20 *μ*M (b, d) AlCl_3_ at 0 and 20 minutes. Various staining patterns signify different cell populations. Region Q3 includes live cells (PI-negative and Annexin V-negative), whereas region Q4 contains apoptotic cells (PI-negative and Annexin V-positive). Dead cells are represented in Q2 region (both PI- and Annexin V-positive), while quadrant Q1 shows damaged cells (PI-positive and Annexin V-negative). Upon a 20-minute exposure to 20 *μ*M AlCl_3_ the cell population from the Q3 region moved to Q1 region, without a clear shift to the Q4 area first. This fact indicates that thymocytes are not undergoing the apoptotic process. Contour plots (e, f) show fluorescence intensity with regard to forward scatter and side scatter in the control (e) and in the presence of 20 *μ*M AlCl_3_ for 20 minutes (f). The increase in forward scatter on exposure to AlCl_3_ is indicative of an increase in size (i.e., swelling).

To determine the nature of the observed cell injuries, we performed experiments which employed the apoptotic detection kit and investigated changes in cell size. Figures 4(a)–4(d) show scattergrams of PI versus Annexin V fluorescence in control and exposed thymocytes. The rationale for this study is that while aluminum does not actually kill thymocytes, it might trigger early events associated with apoptosis. Since Annexin-V detects the movement of phosphatidyl serine to the outer leaflet of the plasma membrane, an increase in Annexin-V fluorescence is indicative of early apoptosis. Region Q3 includes live cells (PI-negative and Annexin V-negative), whereas region Q4 contains apoptotic cells (PI-negative and Annexin V-positive). Dead cells are represented in Q2 region (both PI- and Annexin V-positive), while quadrant Q1 shows damaged cells (PI-positive and Annexin V-negative). Toxicity of aluminum was evident after a very brief exposure resulting in a visible increase in the number of damaged cells (Figure 4(b)). With a 20-minute exposure to 20 *μ*M AlCl_3_, the cell population from the Q3 region moved to the Q2 region, without any shift to the Q4 area, leaving less than half of thymocytes undamaged. This observation indicates that thymocytes are not undergoing an apoptotic process. Rather the shift of cells from the Q3 to the Q1 region suggests damage to these plasma membranes and, if any, a necrotic pathway. Consistent with this conclusion is the result shown in Figures 4(e) and 4(f), which plots side scatter (SSC), a measure of cell granularity, against forward scatter (FSC), which is related to cell size. In the presence of AlCl_3_ (20 *μ*M) for 20 minutes, there is a clear increase in the forward scatter, which indicates an increase in cell size. Necrosis is accompanied by an increase in cell size, whereas apoptosis is associated with cell shrinkage.

## 4. Discussion

Due to its ubiquity, environmental exposure to aluminum may play an important role in the etiology of several diseases [29]. Human ingestion of aluminum from food and beverages represents the major source of intake [30]. It is estimated that the average dietary intake of aluminum in adults ranges from 2 to 3 mg per day. These levels are not considered harmful to people with normal renal function [13]. Dietary exposure is higher in young children and teenagers [31]. However, these exposures do not include intakes associated with the use of personal care products, over-the-counter medication, inhalation of dust, and vaccines. In addition, aluminum becomes more soluble and, thus, even more bioavailable in acidic conditions [32]. Thus, many people with underlying medical conditions are even more vulnerable to aluminum-induced toxicity due to their exposure to higher concentrations of this metal. In other words, total daily aluminum intake by the human body varies broadly and is presumably higher than the levels referenced above [33]. 

In this study we attempted to estimate the range of aluminum concentrations that would be representative of typical daily exposure levels for humans, designating such concentrations as “environmentally relevant”. This is important because even though these are cellular studies, one would hope that the results obtained would be relevant to what would be observed in an intact animal or human. Our results suggest that concentrations of aluminum that would be expected in humans can result in subtle changes in the physiology of immune system cells. While the injury we have observed was seen in acute studies, there may be long-term alterations in immune system function as a consequence. 

Thymocytes were somewhat more sensitive to aluminum toxicity than lymphocytes, exhibiting statistically significant cell injury almost immediately after exposure to 10 *μ*M of AlCl_3_, while lymphocytes showed cell injury only at 15 *μ*M. These results show that while both cell types responsible for immunodefense are quite sensitive to aluminum, thymocytes are somewhat more vulnerable. The reason for this difference is unclear but may reflect their less mature status. 

The injury observed in both thymocytes and lymphocytes was very rapid, occurring in some cases as quickly as measurements could be made. While the mechanism responsible is not clear, the speed of the injury suggests a direct effect on the plasma membrane. This action increases the permeability of the membrane to PI but does not result in total loss of membrane integrity over the period of time we have studied. We conclude that aluminum causes acute damage to the plasma membrane to a degree that allows some entry to PI, but not to such a degree that membrane integrity is completely lost. In acutely isolated cerebellar granule cells, aluminum has been found to cause a rapid neurotic cell death [34] while toxicity of cultured neurons has been reported to induce either apoptosis [35] or a combination of neurosis and apoptosis [36].

Two points are especially important. The concentrations of aluminum studied are environmentally relevant, being ones to which humans are commonly exposed. Secondly, the time course of cell damage was very quick, suggesting a direct damage to the thymocyte/lymphocyte plasma membrane. These results may be relevant to the study and understanding of the mechanism(s) of chronic exposure to low concentrations of aluminum, which may result in long latency and slow progression of disease [23]. In addition, alteration of plasma membrane integrity associated with exposure to aluminum could make cells more permeable to other unwanted substances. Given the prominence of aluminum in the environment and the susceptibility of thymocytes, further investigation of the effects of aluminum on immune system function is warranted.

## 5. Conclusions

We have investigated the immunotoxicological effects of exposure to environmentally relevant concentrations of aluminum. We have documented a dose- and time-dependent injury in murine thymocytes and lymphocytes, which results from exposure to low levels of AlCl_3_ (10 to 40 *μ*M). Less than 5% of thymocytes were damaged after a 60-minute exposure to 10 *μ*M AlCl_3_, while 50% were injured after 10 minutes at 20 *μ*M AlCl_3_. Nearly all thymocytes sustained damage at 30 *μ*M AlCl_3_ after only 5 minutes of incubation. Notable lymphocyte injury was observed at 15 *μ*M AlCl_3_, and less than 50% of cells were injured after a 60-minute exposure to 20 *μ*M. Our data suggest that lymphocytes are less sensitive to aluminum than thymocytes, perhaps due to their more advanced cell maturation. The damage is accompanied by cell swelling, which is consistent with damage to the plasma membrane.
